# Clinical significance and prospective mechanism of increased CDKN2A expression in small cell lung cancer

**DOI:** 10.1007/s12094-023-03376-2

**Published:** 2024-01-11

**Authors:** Dong-Ming Li, Guo-Sheng Li, Jian-Di Li, Feng Chen, Hong Huang, Wan-Ying Huang, Zhi-Guang Huang, Yi-Wu Dang, Yu-Lu Tang, Zhong-Qing Tang, Wen-Jia Tang, Gang Chen, Hui-Ping Lu

**Affiliations:** 1 Department of Pathology, The First Affiliated Hospital of Guangxi Medical University, Guangxi Zhuang Autonomous Region, Nanning, People’s Republic of China; 2 Cardiothoracic Surgery, The First Affiliated Hospital of Guangxi Medical University, Guangxi Zhuang Autonomous Region, Nanning, People’s Republic of China; 3 Medical Oncology, The First Affiliated Hospital of Guangxi Medical University, Guangxi Zhuang Autonomous Region, Nanning, People’s Republic of China; 4 Department of Respiratory and Critical Care Medicine, The First Affiliated Hospital of Guangxi Medical University, Guangxi Zhuang Autonomous Region, Nanning, People’s Republic of China; 5 Department of Pathology, Wuzhou Gongren Hospital, The Seventh Affiliated Hospital of Guangxi Medical University, No.1, Nansanxiang Gaodi Road, Guangxi Zhuang Autonomous Region, Wuzhou, 543000 People’s Republic of China

**Keywords:** Cyclin dependent kinase inhibitor 2A, Small cell lung cancer, Clinical significance, Immunohistochemistry, Kaplan–Meier survival analysis

## Abstract

**Background:**

Although it has been shown that cyclin dependent kinase inhibitor 2A (CDKN2A) plays a significant role in a number of malignancies, its clinicopathological value and function in small cell lung cancer (SCLC) is unclear and warrants additional research.

**Methods:**

The clinical significance of CDKN2A expression in SCLC was examined by multiple methods, including comprehensive integration of mRNA level by high throughput data, Kaplan–Meier survival analysis for prognostic value, and validation of its protein expression using in-house immunohistochemistry.

**Results:**

The expression of CDKN2A mRNA in 357 cases of SCLC was evidently higher than that in the control group (n = 525) combing the data from 20 research centers worldwide. The standardized mean difference (SMD) was 3.07, and the area under the curve (AUC) of summary receiver operating characteristic curve (sROC) was 0.97 for the overexpression of CDKN2A. ACC, COAD, KICH, KIRC, PCPG, PRAD, UCEC, UVM patients with higher CDKN2A expression had considerably worse overall survival rates than those with lower CDKN2A expression with the hazard ratio (HR) > 1.

**Conclusion:**

CDKN2A upregulation extensively enhances the carcinogenesis and progression of SCLC.

**Supplementary Information:**

The online version contains supplementary material available at 10.1007/s12094-023-03376-2.

## Introduction

According to the Global Cancer Statistics 2020 report, with an expected 1.8 million fatalities, lung cancer continues to be the top cause of cancer mortality globally [1–3]. Despite the fact that small cell lung cancer (SCLC) accounts for 15% of cases of lung cancer and non-small cell lung cancer (NSCLC) accounts for 85% [4], SCLCs is more aggressive, which have quicker metastasis and higher growth scores, and more than half of patients are diagnosed at an advanced stage [5]. With a five-year survival rate of < 7%, SCLC is a malignant form of lung cancer that differs from NSCLC in that it has an extremely dismal prognosis. In addition, SCLC is very responsive to chemotherapy, but when it relapses, it has a very high recurrence rate and is nearly always resistant to therapy [6]. Therefore, further research is required to examine possible SCLC indicators and processes in order to develop a theoretical framework for the early detection, diagnosis, and treatment of SCLC.

Oncogenes are often activated during the growth of tumors, whereas tumor suppressor genes are inactivated. It may be a feasible strategy to study the correlation between tumor suppressor genes and SCLC and search for markers. Cyclin dependent kinase inhibitor 2A (CDKN2A) is a cell cyclin dependent kinase inhibitor (CDKI) composed of three exons, which is a tumor suppressor gene [7]. By encoding CDKN2A protein and binding cyclin-dependent kinases (CDKs), CDKN2A inhibits the advancement of the cell cycle from the G1 phase to the S phase, which is essential for the control of cell cycle pathways [8]. CDKN2A mutation inactivation has been reported to be associated with melanoma, NSCLC, head and neck cancers, prostate, esophageal, ovarian, kidney, colon, breast, and bladder cancers. The types of CDKN2A alterations reported in tumors include point reversal, translocation, loss of homozygous or heterozygous, and abnormal promoter methylation [7, 9, 10]. Studies have reported that CDKN2A mutation can lead to the loss of p16 protein encoded by CDK4, resulting in overexpression of CDK4, proliferation of islet B cells, increased insulin secretion, and pancreatic hyperplasia [11]. CDKN2A regulates the expression of cyclinD1 by encoding p16. CyclinD1/CDK4 plays a significant role in cell metabolism and cell cycle progression, indicating that CDKN2A does as well. Chen Y [12] suggests that blocking the cell cycle may be a useful treatment strategy for slowing the spread of pancreatic cancer. However, there has been currently no literature on the comprehensive clinical role of CDKN2A in SCLC, and the precise mechanism of CDKN2A in SCLC is either unclear, therefore further research is necessary.

By examining the expression, clinical implication, and putative molecular mechanism of CDKN2A, this research sought to better understand the mechanism and clinical relevance of CDKN2A in SCLC. The clinical consequence and probable molecular processes of CDKN2A in various malignancies were also explored in this work, revealing the significant function of CDKN2A expression in pan-cancer and offering a fresh and potentially effective therapeutic target.

## Materials and methods

### SCLC public data

Public databases such as ArrayExpress, Gene Expression Omnibus, Oncomine, and The Cancer Genome Atlas (TCGA) were searched for selecting data sets. The search strategies were “lung and (small cell) and (mRNA or gene)”. Inclusion criteria are as follows: (1) The subjects were *Homo Sapiens*; (2) Experiment group samples were SCLC-associated tissues or cells; (3) There were CDKN2A mRNA expression data. Exclusion criteria were as follows: (1) Duplicate or incomplete sample data; (2) The sample number of SCLC group and control group in the integrated data set was less than 3.

### Data processing of SCLC public data

The mRNA expression data of CDKN2A were transformed by log2 (x + 1). To reduce batch effect effects between different datasets, datasets under the same platform (for example, GPL570) were merged. In this procedure, batch effects were reduced using the “SVA” package [13], and gene expression levels in the pooled data sets were standardized using the “limma” [14] or “edge” [15] packages.

As one of the included data sets, GSE30219 also contained the overall survival status, disease-free survival status and follow-up time of SCLC patients, which were collected to perform survival analyses.

### SCLC sample collection and immunohistochemical test from our institute

The First Affiliated Hospital of Guangxi Medical University’s Medical Ethics Review Committee gave its support to this work.

There were 62 tissue samples (26 SCLC tissues and 36 lung tissue control samples).

Tissue sections were subjected to immunohistochemical experiments. Initially, they were dewaxed and repaired. Subsequently, the antigens were extracted by soaking the tissue sections were soaked with 0.01 M citrate buffer (pH = 6.0). The 3%H_2_O_2_ was used to inactivate endogenous peroxidase of tissue sections. Afterward, monoclonal antibody against human CDKN2A was applied to tissue slides (1:100, ab108349, Abcam), and the slides were added with negative management phosphate buffer and were left to incubate at 4 °C overnight. Tissue slices from Shanghai Changdao Biotechnology Co., LTD. in China were stained with 3,3 '-diaminobenzidine and horseradish peroxidase, which was secondary antibody-labeled. Finally, the tissue slices were stained after being dried and sealed before being examined under a microscope. Negative staining was indicated by blue coloration, whereas positive staining was indicated by brown. Two senior pathologists graded the number of positive cells and staining intensity in each sample. The staining intensity score range was 0–3 points, representing no staining, mild staining, moderate staining, and severe staining. The percentage of positive cell score ranged from 0 to 4, representing 5%, 5–25%, 26–50%, 51–75%, and > 75% of positive cells. Staining intensity score and positive cell percentage score represent the protein expression level of CDKN2A.

### Potential function and mechanism of CDKN2A in SCLC

Transcription factors (TFs) play essential roles in regulating certain genes. Therefore, this study explores whether TFs regulate the expression of CDKN2A in SCLC. TFs might regulate CDKN2A expression were obtained from the Cistrome Data Browser, and those with a score greater than 0.9 (indicating a higher likelihood of regulating CDKN2A) were selected. Standardized mean difference (SMD) values of these TFs were calculated to compare their expression between SCLC and non-SCLC groups. The correlation analysis was also performed between the these TFs expression and CDKNK2A expression. TFs were considered to be positively associated with CDKN2A only when expression correlation was detected in at least three (1/4) data sets.

To investigate the putative molecular mechanism of CDKN2A in SCLC, gene set enrichment analysis (GSEA) was carried out using the cluster Profiler program [16]. In this part, the KEGG (Kyoto Encyclopedia of Genes and Genomes) signaling pathways associated with CDKN2A were investigated.

### Significance of CDKN2A in pan-cancer

In order to comprehensively understand the expression patterns and clinical significance of CDKN2A in tumors, a comprehensive pan-cancer analysis on CDKN2A was conducted in this study. Data on TCGA's genome-wide RNA expression came from the Xena database. In this study, peripheral blood samples of primary tumors, normal solid tissues and primary blood-derived cancers were considered for analysis. From the examination of CDKN2A expression and predictive values, tumors with less than 6 samples (3 cancer samples and 3 normal samples) were disqualified. From the Xena database, we extracted the prognostic data types from the TCGA, including overall survival (OS), disease-specific survival (DSS), disease-free interval (DFI), and progression-free interval (PFI).

### Methods of statistics

To find gene expression variations between SCLC and control groups, the Wilcoxon rank sum test and SMD were used. Using the subject’s operating characteristic curve’s area under the curve (AUC), CDKN2A's ability to distinguish SCLC from control samples was assessed. The relationship between CDKN2A expression level and SCLC patient prognosis was investigated using the Kaplan–Meier curve.

All calculation and visualization steps were performed in R software (v4.1.0), except that the integrated subject operating characteristic curve was plotted in Stata 15.0. A *p* value less than 0.05 or 95%CI with SMD without 0 indicated that the analysis results were statistically significant.

## Results

### Overexpression of CDKN2A in SCLC tissues and its prognostic value

In this study, a total of 27 data sets, obtained from 20 research centers and seven countries, were selected. These data sets included 357 SCLC samples and 525 control samples. Further information on the selection of these data sets can be found in Supplementary Material 1.

**Fig. 1:**
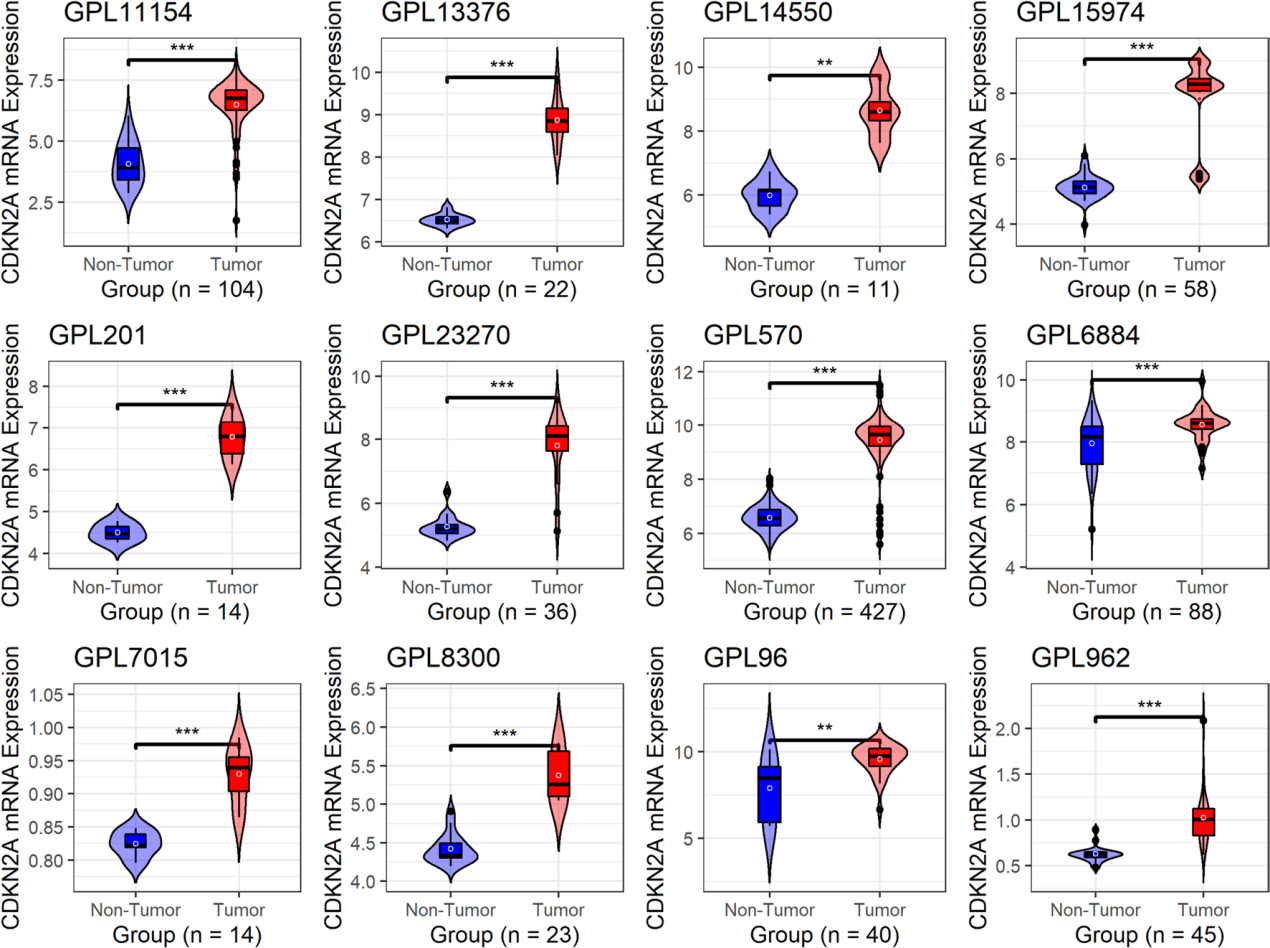
Violin plots of CDKN2A mRNA expression in SCLC. Wilcoxon rank test results are used to obtain the p-value. ^NS^*p* ≥ 0.05; *p < 0.05; **p < 0.01; ***p < 0.001

When compared to the control group, CDKN2A mRNA was substantially elevated in SCLC throughout all 12 combined data sets in our investigation (*p* value of Wilcoxon rank-sum test < 0.05; Fig. 1).

**Fig. 2:**
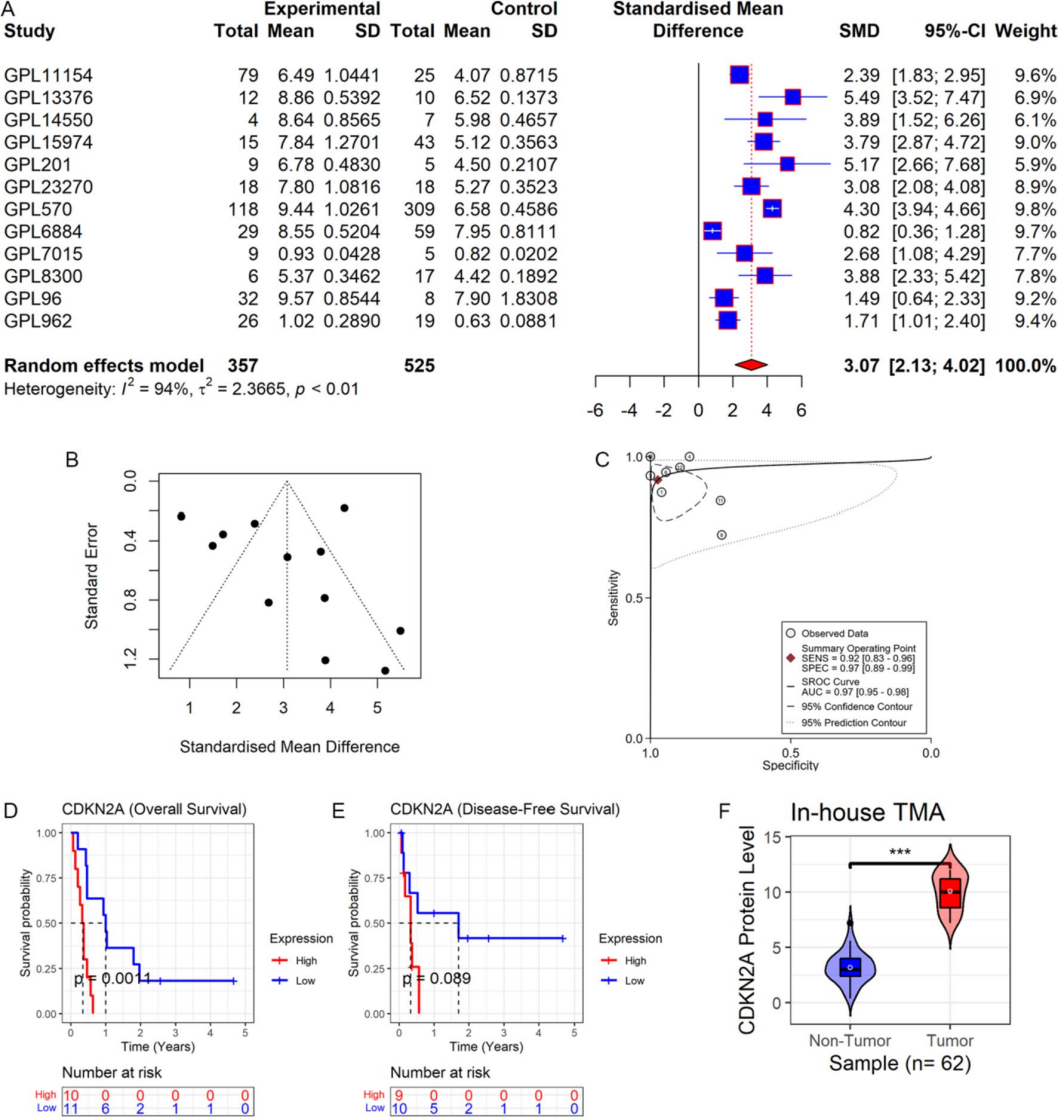
CDKN2A expression and its clinical significance in SCLC. Panel **A**: Forest maps of CDKN2A mRNA expression in SCLC and control tissues. Panel **B**: Publication bias evaluation of SMD (Begg’s *p* = 0.170). Panel **C**: CDKN2A’s ability to identify SCLC samples from non-SCLC samples. Panels **D**, **E**: Association of CDKN2A with prognosis in SCLC patients. Panel **F**: CDKN2A protein levels in SCLC and control groups. *TMA* tissue microarray. P-value was determined using the Wilcoxon rank sum test, ***p < 0.001

The CDKN2A mRNA expression in SCLC (n = 357) was elevated remarkably as compared to the control group [n = 525, SMD = 3.07 (95%CI: 2.13–4.02); Fig. 2A] with no discernible publication bias (*p* = 0.170; Fig. 2B).

### Clinical and prognostic value of CDKN2A in SCLC

As shown in Fig. 2C, CDKN2A showed a significant ability to distinguish SCLC samples from control samples (AUC = 0.97), suggesting that it might be used as a marker to identify SCLC. In terms of prognosis, individuals with SCLC who had high CDKN2A expression had substantially shorter overall survival times than those who had low CDKN2A expression (*p* < 0.05, Fig. 2D), indicating that CDKN2A might be an important prognostic indicator of SCLC patients. However, there was no significant correlation with disease-free survival (*p* > 0.05, Fig. 2E). Furthermore, both univariate and multivariate Cox regression analyses demonstrated that age, tumor stage, and CDKN2A expression were independent prognostic risk factors for patients with SCLC (*p* < 0.05, Table 1).

**Table 1:** Cox regression for clinical paraments and CDKN2A expression of SCLC patients

Category	Univariate Cox regression analysis		Univariate Cox regression analysis	
	Hazard ratio (95% CI)	*p* value	Hazard ratio (95% CI)	*p* value
Age	1.213 (0.456–3.228)	0.700	50.179 (2.081–1209.784)	0.016
Tumor stage	2.184 (1.249–3.818)	0.006	4.075 (1.095–15.159)	0.036
Node stage	1.617 (1.011–2.588)	0.045	1.788 (0.318–10.051)	0.509
Metastasis stage	4.033 (1.181–13.769)	0.026	143.603 (0.627–32,878.268)	0.073
Clinical stage	3.058 (1.453–6.434)	0.003	0.299 (0.014–6.319)	0.438
*CDKN2A*	5.884 (1.787–19.374)	0.004	81.083 (4.271–1539.272)	0.003

### Validation of CDKN2A protein levels in SCLC

**Fig. 3:**
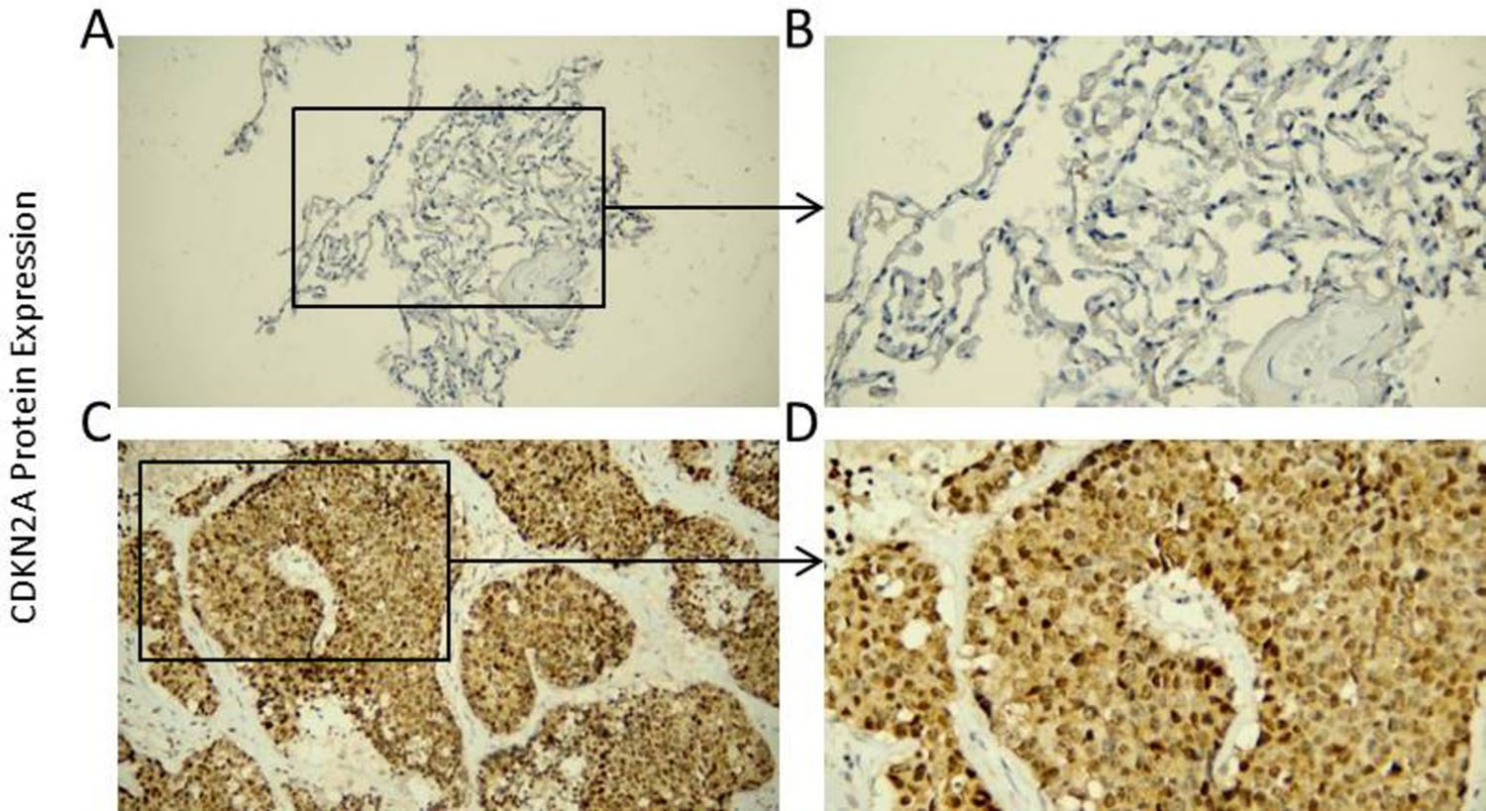
CDKN2A expression in SCLC tissue and control samples under the microscope, the positive sites were located in the nucleus and cytoplasm. Figures **A** and **B** were protein expression level of CDKN2A in control group, figures **C** and **D** were protein level expression of CDKN2A in SCLC. The left figure was 200×, and the right figure was 400×

To confirm the protein level expression of CDKN2A in SCLC tissues, internal samples were analyzed in this study (Fig. 2F). The results demonstrated that the expression level of CDKN2A protein in the SCLC group was greater than that in the control group (*p* < 0.05), which was consistent with the mRNA level. Under the microscope, CDKN2A protein expression in SCLC was evidently higher than in the control group (Fig. 3A–D).

### Potential mechanism of CDKN2A overexpression in SCLC

**Fig. 4:**
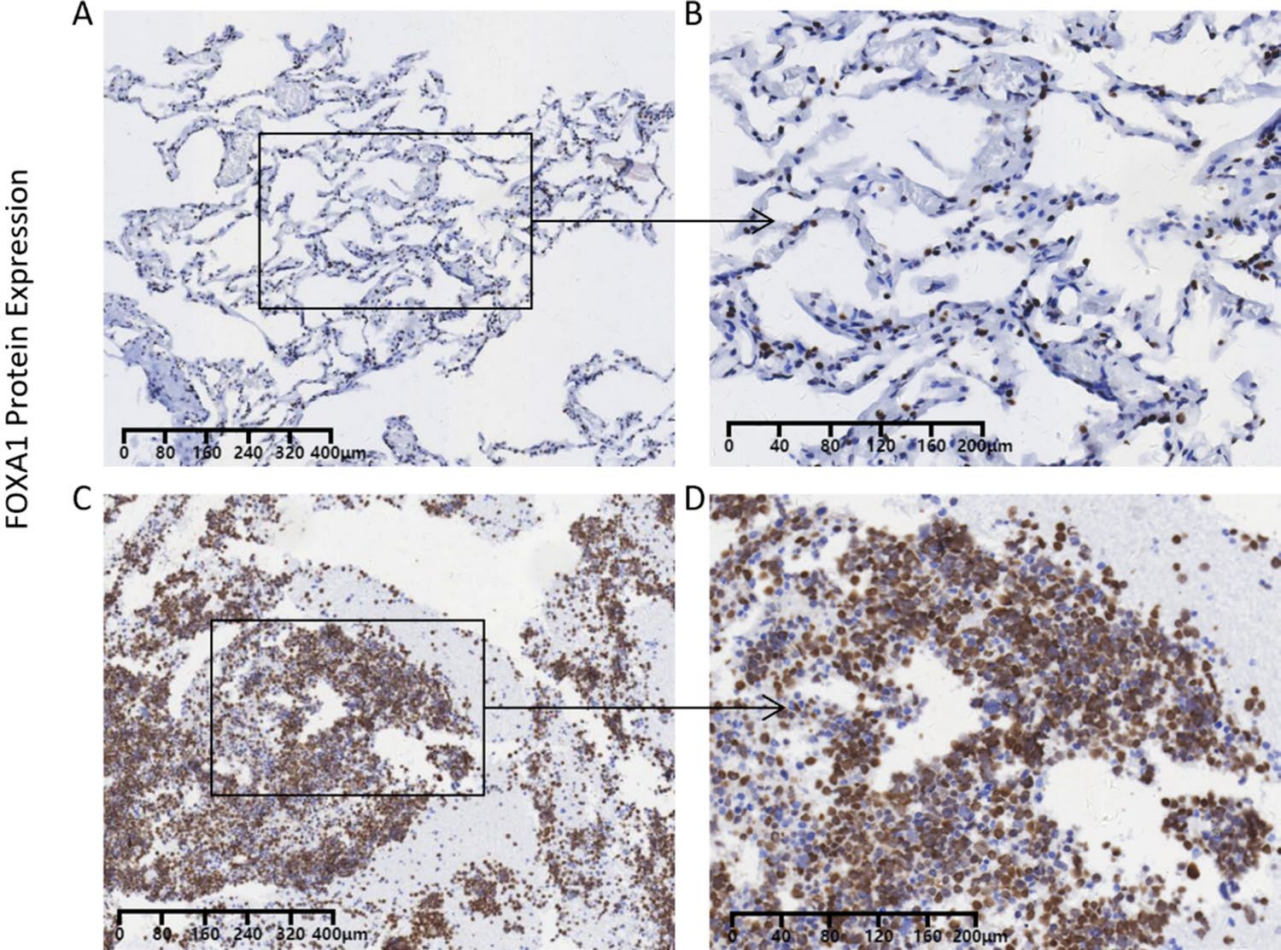
The protein expression of FOXA1 was checked with immunohistochemistry. FOXA1 protein located at nucleus and cytoplasm, was measured in non-cancerous lung tissues (Figure **A** and **B**) and small cell lung cancer (SCLC) (Figure **C** and **D**). The magnification factor of Figure **A** and **C** was 200 × ; Figure **B** and **D** was 400 ×

**Fig. 5:**
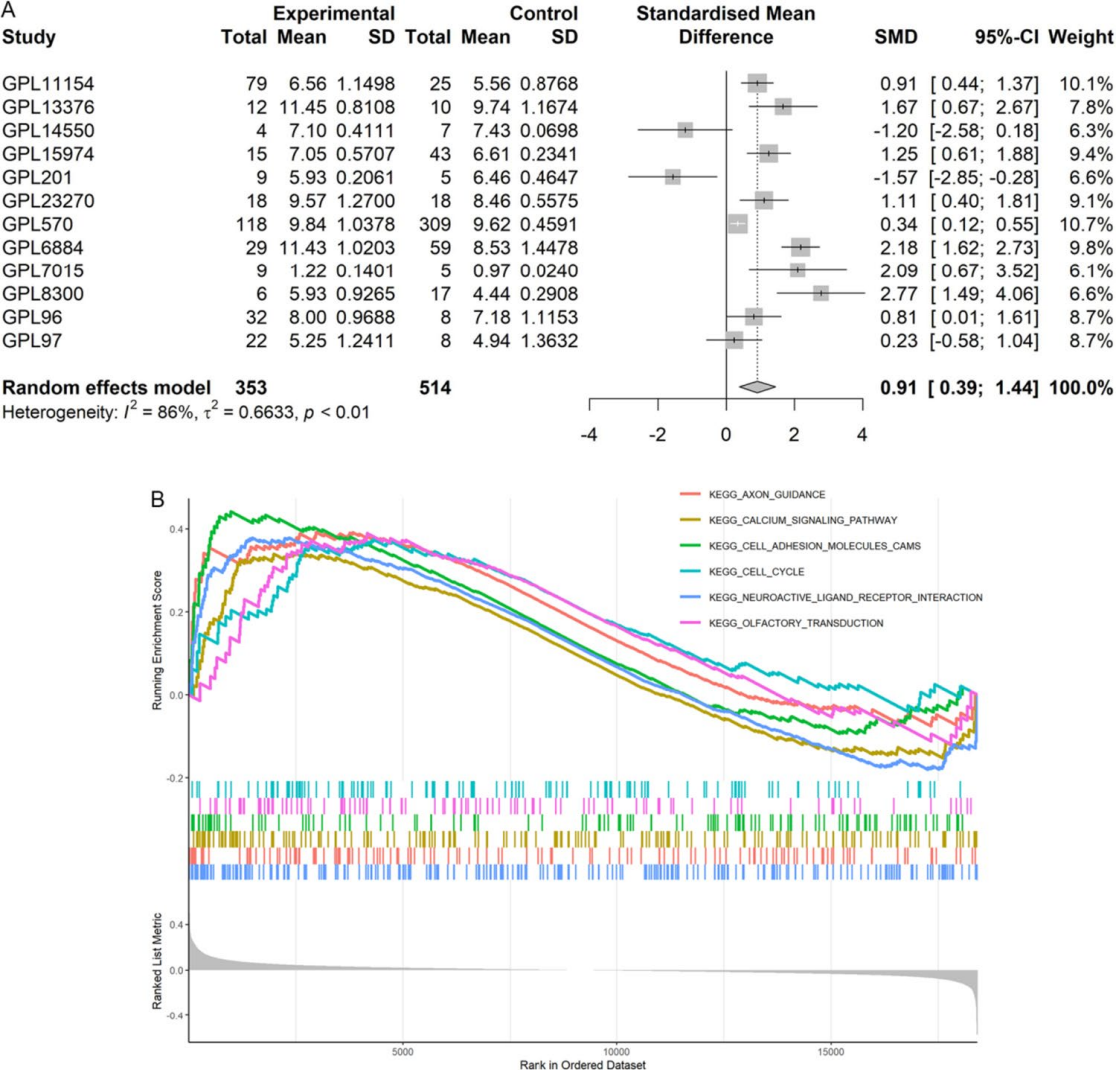
FOXA1 mRNA expression in SCLC and potential molecular mechanism of CDKN2A in SCLC. (**A**) Expression of FOXA1 in SCLC. (**B**) Potential molecular mechanism of CDKN2A in SCLC

Moreover, this study delved into the potential mechanism of the elevated expression of CDKN2A in SCLC. Nine TFs that may regulate CDKN2A expression were obtained from Cistrome Data Browser, including MAX, KDM2B, CTCF, CREBBP, PAX5, FOXA1, SMC1A, H2AZ and CREB1. Differential expression analysis and correlation analysis of the nine TFS showed that FOXA1 was not only highly expressed in SCLC [SMD = 0.91 (0.39–0.44), Fig. 5A), yet at least three data sets indicated that FOXA1 expression in SCLC was positively linked with CDKN2A expression (*p* ≥ 0.3, *p* < 0.05). Furthermore, aim to confirm FOXA1 protein level expression in SCLC tissues, 62 tissue samples (26 SCLC and 33 controls) were studied. The results showed that the expression level of FOXA1 protein in SCLC was significantly higher than that in control group. (Fig. 4). Therefore, increased FOXA1 expression is likely to up-regulate the expression of CDKN2A in the progression of SCLC.

### Molecular mechanism of CDKN2A playing in SCLC

The molecular mechanism of CDKN2A in SCLC remain unknown, which was investigated in this part. The GSEA results demonstrated that CDKN2A may participate in the cell cycle signaling pathway during the emergence of SCLC (Fig. 5B). Additionally, a differential analysis of 39 genes in the cell cycle signaling pathway (excluding CDKN2A) revealed that most of these 39 genes had significantly higher expression levels in the group with high CDKN2A expression compared to the group with low CDKN2A expression, indicating a significant relationship between CDKN2A and these genes (Supplementary material 2). These results suggested that the function of CDKN2A in SCLC might be associated with its typical role in regulating the cell cycle. In addition, CDKN2A might also participate in multiple signaling pathways, among which the first five most significant pathways can be seen in Fig. 5B, indicating that the mechanism of CDKN2A in SCLC may be complex.

### Expression and clinical value of CDKN2A in multiple tumors

**Fig. 6:**
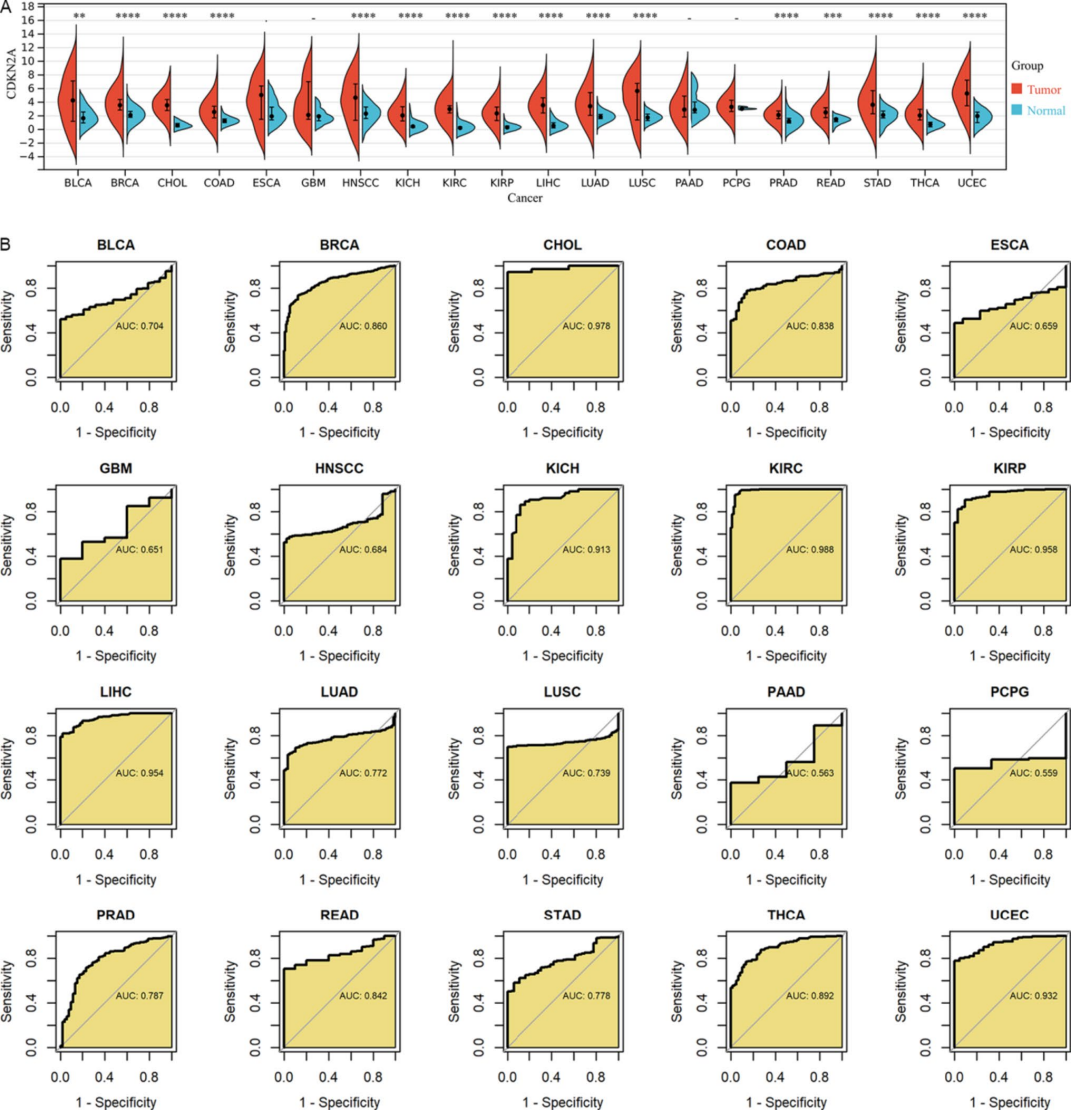
Expression of CDKN2A in a variety of tumors and the ability of CDKN2A to distinguish tumor tissue and control tissue. (**A**) Expression of CDKN2A in various tumor types and the corresponding control populations. (**B**) The ability of CDKN2A to differentiate the tumor tissue from control tissue

Considering that CDKN2A is differentially expressed in SCLC and may play an important role, this study further analyzed the expression and clinical significance of CDKN2A in a number of tumor types. Results revealed that of the 20 tumors examined in this research, 15 tumors showed high levels of CDKN2A expression in cancer tissues compared to control tissues (*p* < 0.05, Fig. 6A).

**Fig. 7:**
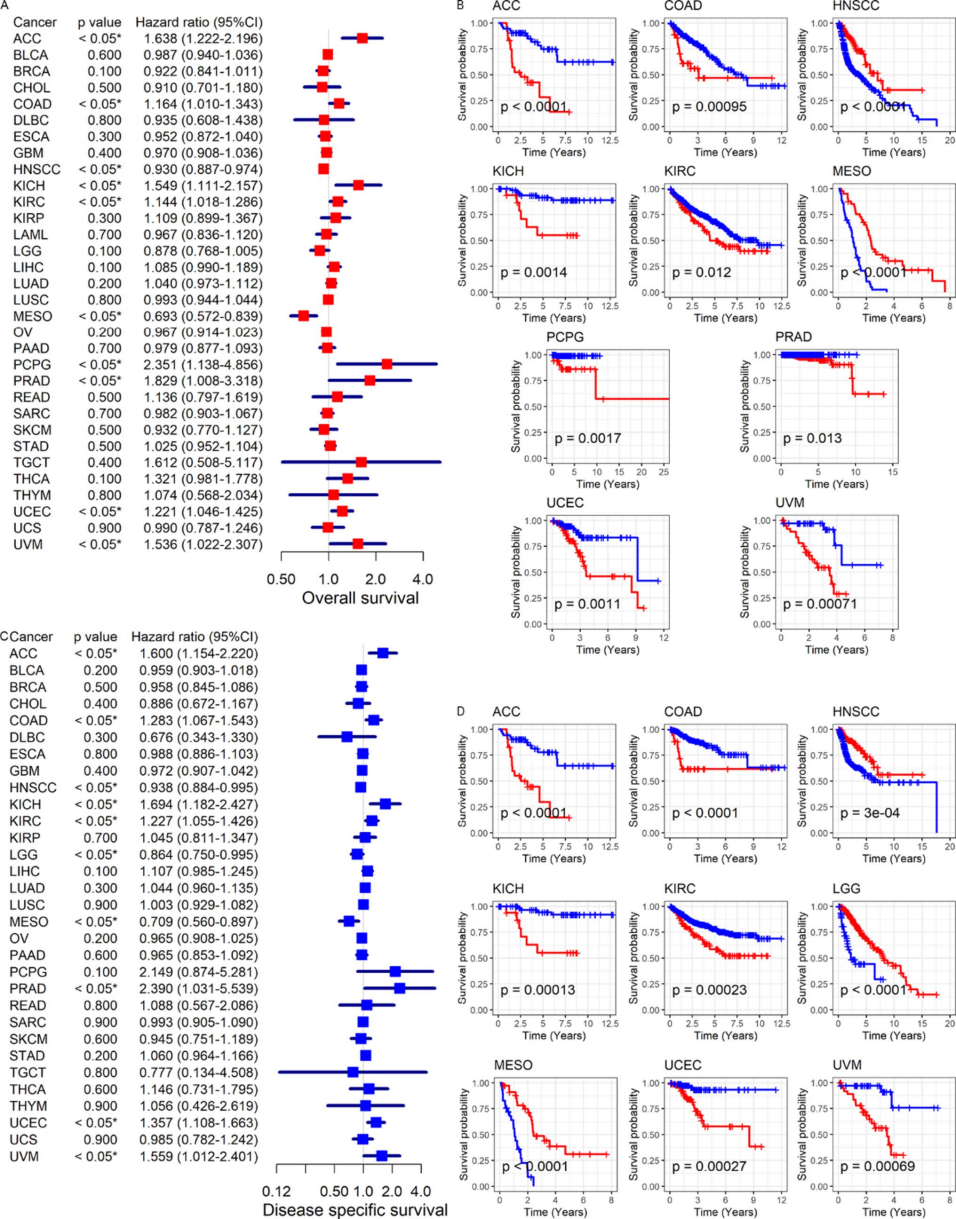
Relationship between CDKN2A expression and cancer patient’s overall and disease-specific survival. (**A**, **B**): Patients with high CDKN2A expression were at a higher risk of developing cancer (**A**) and had a worse chance of surviving their disease (**B**)*.* (**C**, **D**) Patients whose tumors express CDKN2A were at increased risk (**C**), and this correlation was shown to be predictive of a shorter disease-specific survival time (**D**)

**Fig. 8:**
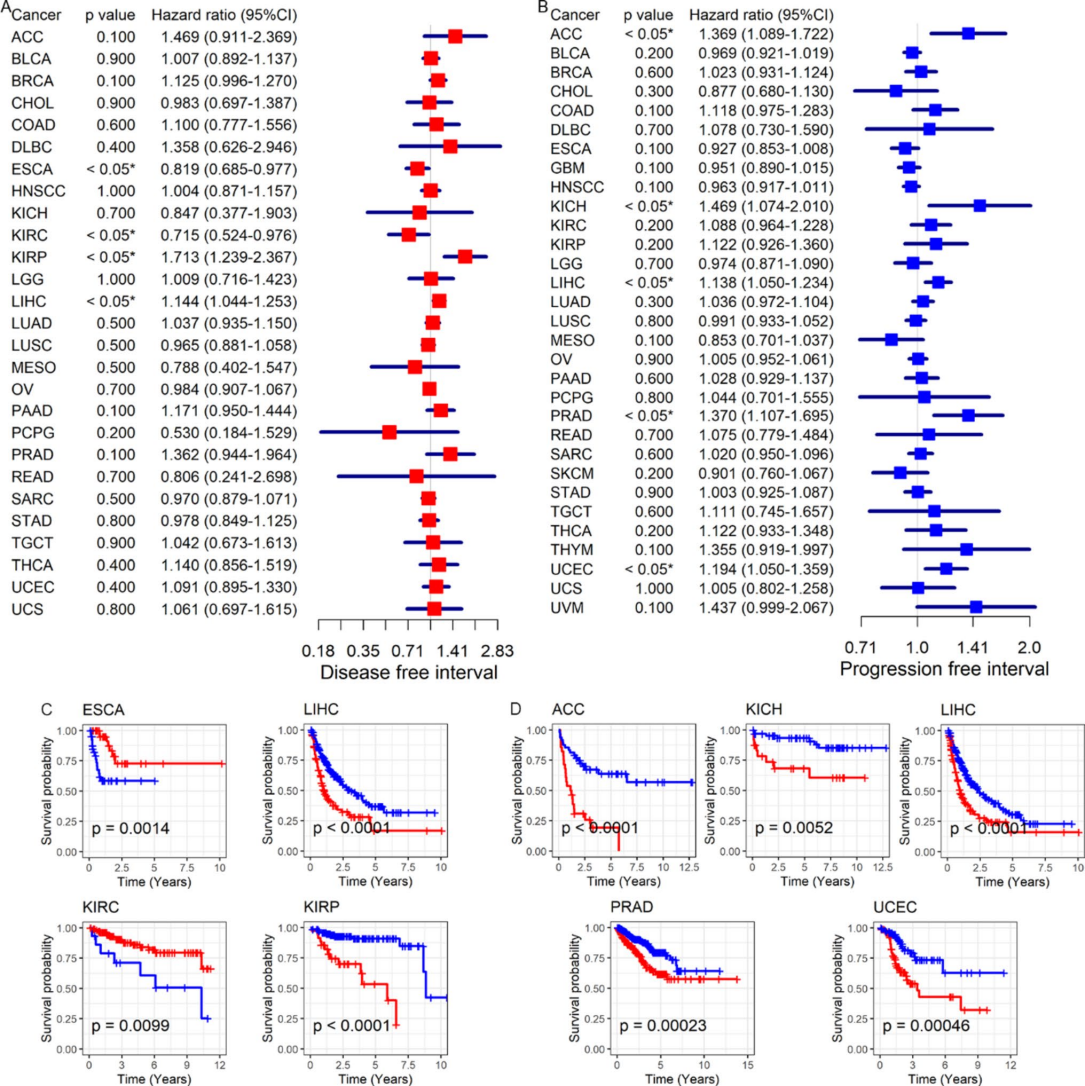
Association between CDKN2A expression and cancer-specific survival and progression-free survival. (**A**, **B**) Shorter disease-free survival time was associated with higher *CDKN2A* expression (**A**), which was a risk factor for some cancer patients (**B**). (**C**, **D**) Patients with high CDKN2A expression were at a higher risk of developing cancer (**C**) and had a shorter progression-free survival time (**D**)

As showed in Fig. 6B, the expression of CDKN2A can distinguish cancer group samples from control group (AUC > 0.75) in a variety of tumor types, figuring that CDKN2A could be a biomarker to locate cancer patients. In terms of prognosis, high expression of CDKN2A might also be used as a prognostic marker for specific tumors. Among them, high expression of CDKN2A was a predictive risk/protective factor for the overall survival of ACC, COAD, KICH, KIRC, PCPG, PRAD, UCEC and UVM patients (*p* < 0.05, Fig. 7A, B), was also a prognostic protective factor for overall survival in HNSCC patients. In addition, CDKN2A expression levels were associated with the prognosis of patients with ACC, COAD, ESCA, HNSCC, KICH, KIRC, KIRP, LGG, LIHC, MESO, PRAD, UCEC, and UVM, at least in disease-specific survival, disease-free survival interval, and progression-free interval (*p* < 0.05, Fig. 7C, D and 8A–D).

### Potential molecular mechanism of CDKN2A in multiple tumors

**Fig. 9:**
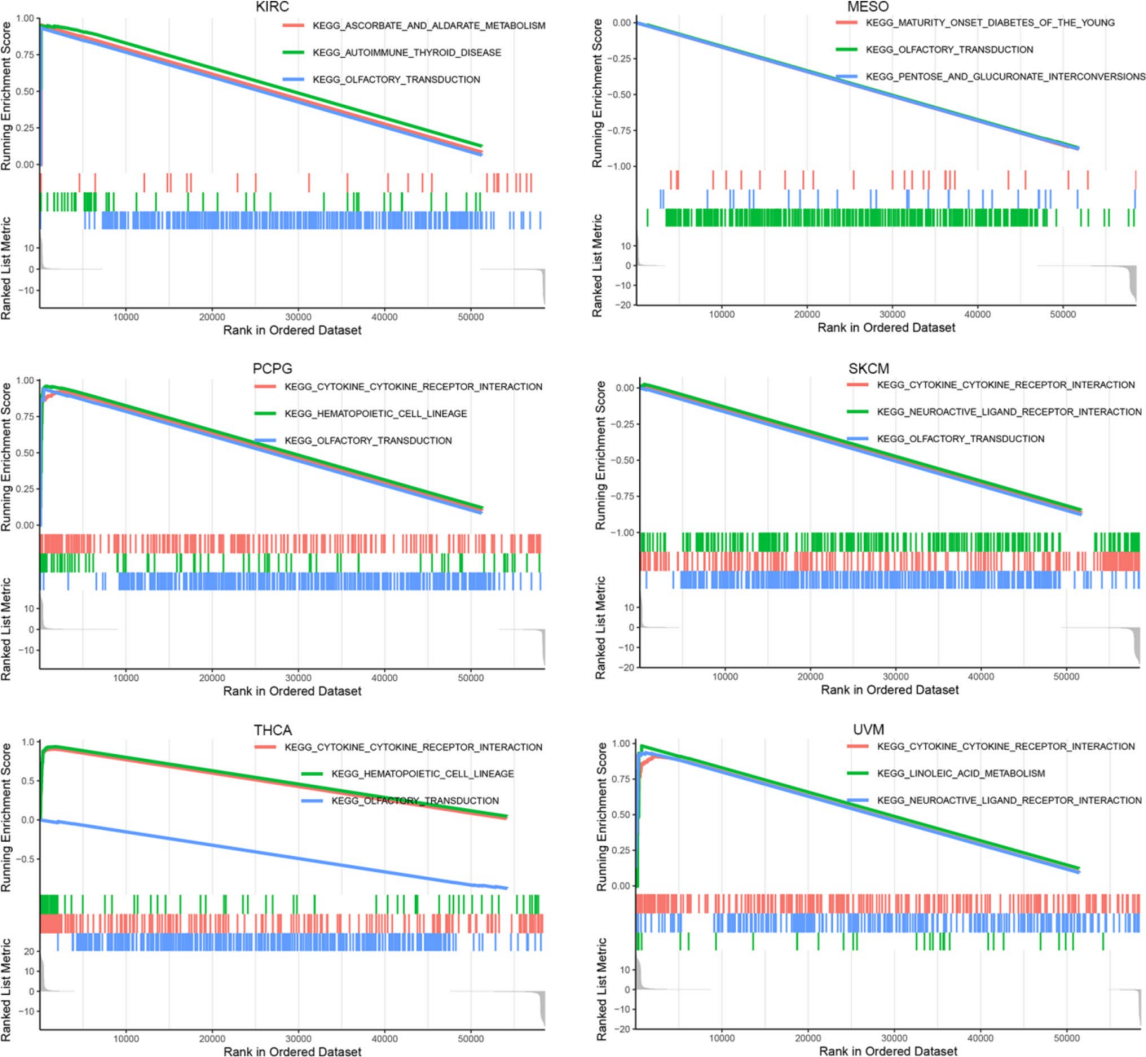
Underlying signaling pathways (Kyoto Encyclopedia of Genes and Genomes) of CDKN2A in a variety of tumors

The exploration of the potential molecular mechanisms of CDKN2A in multiple tumors revealed that CDKN2A was involved in at least three KEGG signaling pathways in six tumors (Fig. 9). Among them, the olfactory transduction pathway and cytokine receptor interaction pathway were the most common (Fig. 9), demonstrating that CDKN2A was participation in olfactory transduction pathway and cytokine receptor interaction pathway might have significant effects on the formation and growth of several cancers.

## Discussion

Although there are more and more studies on CDKN2A, the literature on CDKN2A in SCLC is rarely reported, and the exact mechanism of CDKN2A in SCLC has not yet been identified. Therefore, in this work, the high expression of CDKN2A in SCLC was identified by analyzing all 12 included combined data sets and internal samples, which required the utilization of big samples from a variety of sources. CDKN2A could be used as a marker for SCLC identification, revealing the significant prognostic and identification value of CDKN2A in SCLC. We also investigated the role of CDKN2A in SCLC and discovered that its overexpression was linked to FOXA1. In addition, we studied the role of CDKN2A in pan-carcinoma, and the findings suggested that CDKN2A might be utilized as a marker for selecting potential cancer patients for further study, and high expression of CDKN2A might also be used as a prognostic marker for specific tumors, and CDKN2A had important clinical value in a variety of tumors.

Regulation of the cell cycle and growth control are two of primary functions of CDKN2A, and its encoded product is cyclin-dependent kinase 4(CDK4), which prevents cells from developing to S phase by inhibiting CDK4. Studies have shown that when CDKN2A is deficient or its expression is downregulated, the cell cycle will be out of control, cell proliferation will be accelerated, and the cells will be malignant [17]. Head and neck squamous cell carcinoma has a loss rate of CDKN2A that may exceed 80%, and esophageal cancer, ovarian cancer, and pancreatic cancer all show evidence of homozygous loss of CDKN2A [18]. In addition, CNKN2A has been shown to be up-regulated in a number of tumor tissues, with this up-regulation correlating with clinical features and patient prognosis [19]. This research utilized a large sample size from a variety of sources and pooled 12 data sets because of the potential significance of variable CDKN2A expression in malignancy. In the meanwhile, the random effects model was built to compute SMD for a unified study of CDKN2A mRNA expression levels across 12 datasets. A high level of CDKN2A mRNA expression was seen in SCLC. In addition, this study also verified the expression of CDKN2A in SCLC tissues from the protein level, and analyzed internal samples. Consistent with the findings at the mRNA level, the data demonstrated that the protein expression level of CDKN2A was higher in SCLC. The data from the experiments suggested that CDKN2A played a role in the incidence and development of SCLC.

Multiple types of cancers have been linked to CDKN2A in scientific studies. Researchers have shown that aberrant expression of CDKN2A is linked to the onset of malignant tumors such lung cancer, breast cancer, bone tumors, and skin cancer when homozygous deletion of CDKN2A is present [10, 20, 21]. In addition, studies have found that pancreatic cancer tumor growth and proliferation are related to CDK2/4/6 activation caused by CDKN2A deletion, and CDKN2A is closely related to the malignancy of esophageal squamous cell carcinoma [22]. The methylation of CDKN2A may be an early molecular event in the occurrence of esophageal squamous cell carcinoma [23]. CDKN2A inactivation is linked to the development of cancer via three primary mechanisms: suppression of RNA polymerase activity, alteration of chromatin structure, and prevention of transcription factor binding to DNA [24]. These results further strengthen the link between CDKN2A expression and the onset and progression of malignancies. The diagnosis and treatment of SCLC are greatly aided by a thorough understanding of the molecular process behind the overexpression of CDKN2A in SCLC tissues. Through differential expression analysis and correlation analysis of 9 TFs that might regulate CDKN2A expression obtained from Cistrome Data Browser in this study, it was found that FOXA1 was not only highly expressed in SCLC, but also positively correlated with CDKN2A expression in SCLC. Therefore, increased FOXA1 expression is likely to up-regulate the expression of CDKN2A in the progression of SCLC. GSEA was performed by taking the median expression value of CDKN2A as the truncation value. The findings imply that CDKN2A could participate in the cell cycle signaling pathway during the development of SCLC. The expression of the majority of these 39 genes was significantly upregulated in the group with high CDKN2A expression compared to the group with low CDKN2A expression, demonstrating a significant association between CDKN2A and these genes, according to additional differential analysis of 39 genes in the cell cycle signaling pathway. These findings implied a potential involvement for CDKN2A in the control of the cell cycle. Further evidence that the mechanism of CDKN2A in SCLC may be complicated comes from the possibility that it participates in a number of signaling pathways.

Previous research has shown a strong correlation between the clinical characteristics and prognosis of lung cancer patients and the degree of CDKN2A expression in lung cancer tissues [25]. It was discovered that the CDKN2A gene's mutation rate in lung cancer tissues was greater than that in nearby tissues, and that patients with CDKN2A mutations had higher recurrence rates than patients with wild-type CDKN2A. Lung cancer diagnosed as CDKN2A mutant is closely connected [26]. Those with CDKN2A mutations had a worse 3-year survival rate than patients with wild-type CDKN2A. Studies have demonstrated that the CDKN2A gene mutation in patients with lung cancer has diagnostic significance for lung cancer and is a separate risk factor impacting the patients' postoperative survival [27]. The clinical significance of elevated CDKN2A expression in SCLC patients is still unknown since there have been few research reporting CDKN2A expression in SCLC. In this study, CDKN2A was found to be significantly effective in distinguishing SCLC group samples from control group samples, suggesting that CDKN2A may be used as a marker for SCLC identification. While there was no significant link between CDKN2A and disease-free survival, the overall survival of SCLC patients with high CDKN2A expression was considerably worse than that of patients with low CDKN2A expression, suggesting that CDKN2A may be a key prognostic indicator for SCLC patients. Additionally, it has been shown that CDKN2A mutations that cause loss of function in somatic cells are linked to lung, head and neck, gastroesophageal, melanoma, stomach, colon, and non-small cell lung cancers, indicating that CDKN2A is involved in a number of tumor types [28]. There hasn’t been any research on CDKN2A in all types of cancer, however. Our findings in this research showed that CDKN2A might be utilized as a marker to identify cancer patients as it could be used to differentiate between cancer tissue samples and control groups in a variety of tumor forms. High expression of CDKN2A might also serve as a prognostic marker for specific tumors. One of them was the high expression of CDKN2A, which was highly connected with patient overall survival and was a predictive risk/protective factor for the overall survival of ACC, COAD, KICH, KIRC, PCPG, PRAD, UCEC, UVM patients, was also a prognostic protective factor for overall survival in HNSCC patients. In addition, CDKN2A expression levels were associated with the prognosis of patients with ACC, COAD, ESCA, HNSCC, KICH, KIRC, KIRP, LGG, LIHC, MESO, PRAD, UCEC in disease-specific survival, disease-free survival interval, and progression-free interval patients.

Considering that CDKN2A was differentially expressed in SCLC and might play an important role, this study further analyzed the expression and potential molecular mechanism of CDKN2A in a number of tumor types. The outcomes of this study’s investigation indicated that CDKN2A expression difference was observed between cancer tissues and control tissues of 15 kinds of tumors, and all showed high expression. The potential molecular mechanism of CDKN2A in various tumors was explored, and CDKN2A was found to be involved in at least 3 KEGG signaling pathways in 6 tumors. Among them, the olfactory transduction pathway and cytokine receptor interaction pathway was the most common, suggesting that through its participation in in these two key signaling pathways, CDKN2A might have a significant impact on the incidence and progression of different cancers.

## Conclusion

In summary, we discovered that CDKN2A had a high expression in SCLC and had significant therapeutic relevance. Some malignancies, including SCLC, might benefit from the use of CDKN2A as a biomarker for diagnosis and therapy.

### Supplementary Information

Below is the link to the electronic supplementary material.

Supplementary file1 (DOCX 325 KB)

Supplementary file2 (DOCX 20 KB)

## Data Availability

Datasets from public databases can be obtained from SangerBox (http://vip.sangerbox.com/), ArrayExpress (https://www.ebi.ac.uk/arrayexpress/), GEO (https://www.ncbi.nlm.nih.gov/gds/?term =), GTE-x (https://www.gtexportal.org/home/index.html), and the TCGA Research Network (www.cancer.gov/tcga) with dataset IDs (e.g., GSE32036). Data contained in in-house tissues can be obtained from the corresponding author.
