# Supplementary material for: Clinical significance and prospective mechanism of increased CDKN2A expression in small cell lung cancer
Source: Clin Transl Oncol. 2024 Jan 11;26(6):1519–31. doi: 10.1007/s12094-023-03376-2 (PMC11108933; doi:10.1007/s12094-023-03376-2)
Supplement: Supplementary file 1 — Supplementary file1 (DOCX 325 KB) [file 12094_2023_3376_MOESM1_ESM.docx]

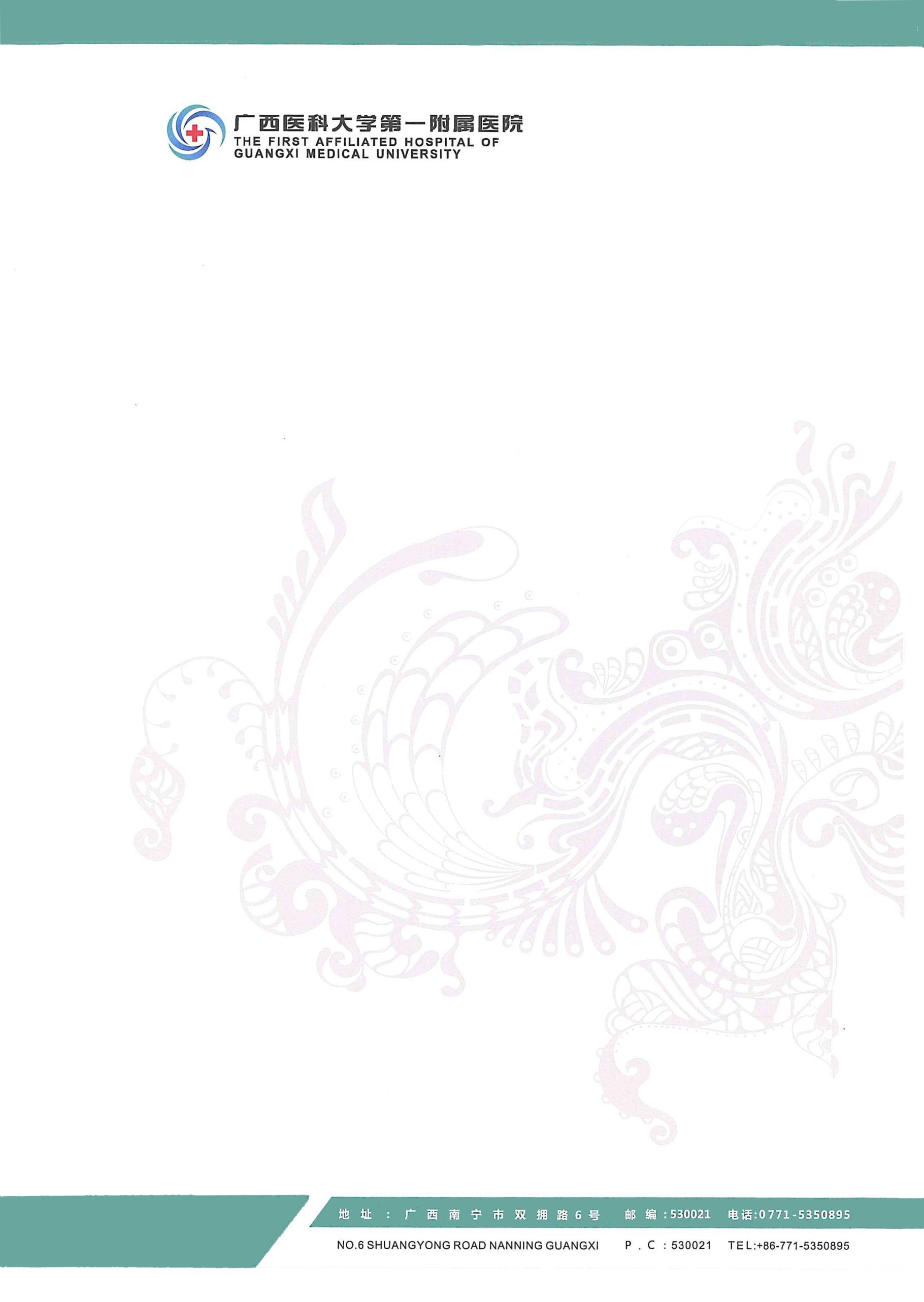


# FIRST AFFILIATED HOSPITAL of GUANGXI MEDICAL UNIVERSITY

**ETHICAL REVIEW COMMITTEE Approval Notice**

**Approval Number:** 2023-E691-01

**Title:** Article title: Clinical Significance and Prospective Mechanism of Increased CDKN2A Expression in Small Cell Lung Cancer

**Research Contents:** Background: Although it has been shown that cyclin dependent kinase inhibitor 2A (CDKN2A) plays a significant role in a number of malignancies, its clinicopathological value and function in small cell lung cancer (SCLC) is unclear and warrants additional research.

Methods: The clinical significance of CDKN2A expression in SCLC was examined by multiple methods, including comprehensive integration of mRNA level by high throughput data, Kaplan-Meier survival analysis for prognostic value, and validation of its protein expression using in-house immunohistochemistry.

Results: The expression of CDKN2A mRNA in 357 cases of SCLC was evidently higher than that in the control group (n=525) combing the data from 20 research centers worldwide. The standardized mean difference (SMD) was 3.07, and the area under the curve (AUC) of summary receiver operating characteristic curve (sROC) was 0.97 for the overexpression of CDKN2A. ACC, COAD, KICH, KIRC, PCPG, PRAD, UCEC, UVM

patients with higher CDKN2A expression had considerably worse overall survival rates than those with lower CDKN2A expression with the hazard ratio (HR) ＞1.

Conclusion: CDKN2A upregulation extensively enhances the carcinogenesis and progression of SCLC.

**Applicant:** Dong-Ming Li

**Application Department:** Pathology department

**Date of Application:** December 07, 2023

**Date of Approval:** December 07, 2023

**Conclusion:** This paper fully considered and protected the rights and interests of the study objects. It meets the criteria of Ethical Review Committee. The Medical Ethics Committee of First Affiliated Hospital of Guangxi Medical University has approved the

protocol.

Signature:


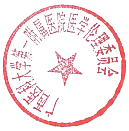

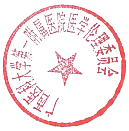

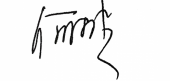


(Vice) Director of Ethical Review Committee

First Affiliated Hospital of Guangxi Medical University Date: December 07, 2023
