# Supplementary material for: Clinical significance and prospective mechanism of increased CDKN2A expression in small cell lung cancer
Source: Clin Transl Oncol. 2024 Jan 11;26(6):1519–31. doi: 10.1007/s12094-023-03376-2 (PMC11108933; doi:10.1007/s12094-023-03376-2)
Supplement: Supplementary file 2 — Supplementary file2 (DOCX 20 KB) [file 12094_2023_3376_MOESM2_ESM.docx]

| CDKN2A and FOXA1 Protein Expression in 62 Tissue Samples Score in SCLC | | | | |
| --- | --- | --- | --- | --- |
| ID | Gender | Age | CDKN2A protein score | FOXA1 protein score |
| N1 | Male | 67 | 7.2 | 5.2 |
| N2 | NA | NA | 2.8 | 4.1 |
| N3 | Female | 65 | 2.4 | 1.5 |
| N4 | NA | NA | 3.6 | 3.8 |
| N5 | Male | 65 | 3.6 | 0.8 |
| N6 | Male | 66 | 5.6 | 6.4 |
| N7 | Male | 71 | 2.4 | 5.7 |
| N8 | Male | 48 | 0.4 | 3.5 |
| N9 | Male | 63 | 1.2 | 2 |
| N10 | Male | 55 | 1.2 | 1.2 |
| N11 | Male | 56 | 2.4 | 2.5 |
| N12 | Male | 68 | 2.4 | 3.1 |
| N13 | Male | 54 | 2.8 | 2.5 |
| N14 | Male | 59 | 2.8 | 2 |
| N15 | Male | 72 | 4 | 2 |
| N16 | Male | 71 | 4 | 3 |
| N17 | Male | 47 | 4 | 2.2 |
| N18 | Male | 77 | 4 | 1.1 |
| N19 | Male | 77 | 4.4 | 0.8 |
| N20 | Male | 55 | 5.2 | 0.5 |
| N21 | Male | 65 | 5.6 | 3.1 |
| N22 | Male | 55 | 0.8 | 4 |
| N23 | Female | 65 | 1.6 | 2.8 |
| N24 | Male | 48 | 2 | 0.6 |
| N25 | Male | 52 | 2.4 | 0.8 |
| N26 | NA | NA | 2.4 | 1.6 |
| N27 | Male | 56 | 2.4 | 2.4 |
| N28 | Male | 65 | 2.8 | 2 |
| N29 | Male | 77 | 2.8 | 2 |
| N30 | Male | 72 | 3.2 | 3.5 |
| N31 | Male | 77 | 3.2 | 4.5 |
| N32 | NA | NA | 3.6 | 1.9 |
| N33 | Male | 65 | 3.6 | 1.5 |
| N34 | Male | 59 | 4 | 3 |
| N35 | Male | 47 | 4 | 2.2 |
| N36 | Male | 66 | 4.4 | 5.2 |
| T1 | Male | 67 | 11.2 | 9 |
| T2 | NA | NA | 7.2 | 12 |
| T3 | Female | 65 | 7.6 | 10.5 |
| T4 | NA | NA | 7.6 | 7 |
| T5 | Male | 65 | 8 | 8.5 |
| T6 | Male | 66 | 8 | 13 |
| ID | Gender | Age | CDKN2A protein score | FOXA1 protein score |
| T7 | Male | 71 | 8 | 11.5 |
| T8 | Male | 48 | 12 | 10.5 |
| T9 | Male | 63 | 11.2 | 8 |
| T10 | Male | 55 | 11.2 | 12 |
| T11 | Male | 56 | 10 | 13.2 |
| T12 | Male | 68 | 12 | 12.5 |
| T13 | Male | 54 | 10 | 11.2 |
| T14 | Male | 59 | 12 | 10 |
| T15 | Male | 72 | 9.6 | 8 |
| T16 | Male | 71 | 9.6 | 12 |
| T17 | Male | 47 | 11.2 | 11.5 |
| T18 | Male | 77 | 12 | 14 |
| T19 | Male | 77 | 9.2 | 13.5 |
| T20 | Male | 55 | 12 | 10.8 |
| T21 | Male | 65 | 11.2 | 12.3 |
| T22 | Male | 51 | 8.4 | 10 |
| T23 | Male | 58 | 9.2 | 12.3 |
| T24 | NA | NA | 10 | 9 |
| T25 | Male | 55 | 11.2 | 12.4 |
| T26 | Male | 57 | 12 | 12.5 |
